# Polar Wax as Adhesion Promoter in Polymeric Blend Films for Durable Photovoltaic Encapsulants

**DOI:** 10.3390/ma15196751

**Published:** 2022-09-29

**Authors:** Marilena Baiamonte, Elisabetta Morici, Claudio Colletti, Nadka Tz. Dintcheva

**Affiliations:** 1 Dipartimento di Ingegneria, Università di Palermo, Viale delle Scienze, Ed. 6, 90128 Palermo, Italy; 2 ATeN Center, Università di Palermo, Viale delle Scienze, Ed. 18, 90128 Palermo, Italy; 3 Enel Green Power SpA Contrada Blocco Torrazze, Zona Industriale Catania, 95121 Catania, Italy

**Keywords:** photovoltaic, polymeric encapsulant, EVA, crosslinking agent, stabilizing system, adhesion promoter

## Abstract

Technological developments in the solar photovoltaic field must guarantee the high performance and low deterioration of solar cells in order for solar power plants to be more efficient and competitive. The solar cell needs comprehensive protection offered by a polymeric encapsulant, which improves UV stability, reduces water and moisture absorption, reduces oxygen and vapor permeability and enhances mechanical resistance. Moreover, high transparency and adhesion yields improved the solar panel performance. The current work analyzes polymeric films based on poly(ethylene-co-vinyl acetate) (EVA) and polyolefin (PO) for photovoltaic encapsulant use (the high temperature resistance is improved by adding PO to EVA, as investigated and documented before). To enhance the mechanical resistance and optical properties of the investigated matrices, a crosslinking agent, an adhesion promoter and stabilizing agents have been incorporated in both EVA and EVA/PO systems. The adhesion promoter is a polar wax–silane-free agent; the absence of the silane function allows the integrity of the module to be maintained over time. All samples were characterized through mechanical and rheological analysis, and their long-term UV stability was investigated by accelerated ageing and by FTIR and UV–vis spectroscopy. The obtained results suggest that the presence of a crosslinking agent, an adhesion promoter and stabilizers in EVA/PO-based films allows for the achievement of the required features for the encapsulants, showing mechanical and rheological behavior similar to those of EVA containing the same additives.

## 1. Introduction

The growing socio-political concern over environmental issues highlights the need for a green energy transition and urgent action on a global scale to limit climate change. The decarbonization of the energy sector, necessary to reduce CO_2_ emissions, leads to replacing fossil fuel-generated electricity with energy produced from renewable sources. Solar energy is the cleanest and fastest-growing renewable energy source, with its worldwide capacity increasing by up to 650 GW in the last 10 years, which can be leveraged using different technologies, such as solar heating, solar photovoltaic and solar thermal electricity.

Photovoltaic (PV) panels convert the sun’s energy into electrical energy, and their conversion efficiency, durability and stability are key factors for their development and market penetration. The current operating life of a PV module is less than 25 years. The latest generation of double-sided heterojunction photovoltaic panels, produced by 3SUN (ENEL Green Power, Rome, Italy), can maintain high properties and performance for about 35–40 years [1].

Advances in technological innovation, especially in the material science area, would further reduce costs and extend the durability of PV panels. Therefore, research efforts are focused on the optimization of the encapsulation process to bring costs down and on testing encapsulant polymeric materials to enhance long-term stability. The encapsulation technique, indeed, is reputed as an effective method to efficiently protect the active PV elements and to ensure their reliability and performance, improving the operational stability under ambient conditions of the PV module [2,3,4]. In other words, the optimized encapsulation films also provide the opportunity to realize greater efficiency and durability in the module manufacturing process.

The main characteristics of an encapsulant material are processability, flexibility, mechanical integrity at working temperatures, thermal and electrical insulation, high transmittance of light and resistance to moisture and oxygen penetration. Moreover, a long-term stability to UV exposure and a strong adhesion between the layers of the module are also required to avoid discoloration, premature delamination and corrosion of the PV module [5,6,7,8].

The chosen materials for encapsulant films have changed over time. For example, polydimethylsiloxane (PDMS) was first used as a photovoltaic encapsulant due to its flexibility, relatively low viscosities at molecular weights high enough to avoid high volatility, thermal stability, optical properties and UV exposure stability, but the high cost of PDMS makes it unsuitable for PV panel applications.

Currently, the most widely used photovoltaic encapsulant material is low-melting EVA, which shows a good balance between performance and cost [9]. Other usual materials employed as protective layers and encapsulant protective layer materials in solar modules are polyvinyl butyral (PVB) and thermoplastic polyolefin (TPO). PVB provides strong binding and adhesion properties with glass, transparency and flexibility, but also water uptake and high sensitivity to hydrolysis. The TPO structure consists of a polyethylene backbone with different side groups, such as acrylates, acrylic acids and alkanes, replacing the vinyl acetate moieties of EVA. Therefore, during degradation, TPO cannot produce acetic acid, which accelerates the oxidation process and could cause yellowing and corrosion. At the moment, however, TPO’s thermal degradation and stability in environmental conditions, as well as long-term stability, are still under investigation.

For all polymeric materials that are unmixed, blended or combinations of films, it is important to underline that good adhesion between the module layers ensures the efficiency of the solar cells. Interlayer delamination, i.e., a partial or an entire delamination of the layers from an adjacent layer, and weak interfaces can lead to voids that could become weakness zones for moisture, potentially leading to corrosion of the metal contacts [10,11,12]. Therefore, in order to have suitable performance, trialkoxy silanes are often utilized to promote adhesion between the polymers, with particular regard to poly(ethylene-co-vinyl acetate), EVA and inorganic surfaces [13]. The silane end groups could react with hydroxyl groups, forming interfacial structures that ensure proper adhesion and help passivate inorganic surfaces against corrosion. The drawbacks of silanes are related to their thermal degradation and degradation in service that, in turn, leads to delamination of the modules [10,14,15] and their lower efficiency [16,17]. Thus, a new kind of adhesion promoter needs to be found and tested. Furthermore, for optimizing material performance, it must be considered that discoloration, moisture absorption, corrosion, acetic acid formation, crosslinking reactions, bubble formation and delamination could occur in EVA-based encapsulants [18]. Additives such as antioxidants and UV absorbers must be added imperatively [19,20]. Moreover, EVA requires peroxide crosslinking agents to obtain strong and stable laminates. An interesting review reported on the status of considering materials for organic and perovskite solar cell encapsulants [21]. Therefore, the search for new better-performing materials for more efficient energy recovery continues, and, interestingly, the formulation of flexible perovskite solar cells using bioinspired liquid-repelling sealing films has been documented [22].

To additionally increase the properties of encapsulants, EVA could be partially substituted with a fraction of high-performance polyolefins. In our previous study, the possibility of a partial replacement of EVA with a thermoplastic polyolefin (PO) was investigated and documented [23]. The research established that the best results, in terms of properties and performance, are obtained for the blend of 75/25 weight percentages for EVA and PO, respectively (EVA/PO = 75/25 wt.%). The beneficial effect in the formulation of EVA/PO blends is mainly related to the production of PV encapsulant films that have an improved thermo-oxidative resistance at high temperatures, making the encapsulant films more resistant to accidental hot-spot temperature increases, which is the main reason for PV failure. The second reason for PV encapsulant failure, especially at high temperatures, is related to the formation of degradation products coming from the degradation of EVA, i.e., the formation of acetic acid, and from the degradation of silane sealants, which leads to the formation of volatile molecules. Therefore, the EVA/PO blend encapsulant films that contain silane-free adhesion promoters could be considered good candidates for the formulation of PV modules that reach high temperature values in service.

In this work, encapsulant films based on EVA and EVA/PO blends with and without a crosslinking agent, a UV absorber, antioxidants, a metal deactivator and a commercial adhesion promoter (here referred to as TEG) containing no silane agents were prepared by melt mixing, and their performance was accurately compared. Therefore, since the photo-oxidative resistance study is a significant parameter for predicting the lifetime and long-term performance of PV modules, all samples were subjected to accelerated exposure to UVB rays, and the degradation trends of EVA and EVA-based samples, as well as EVA/PO and EVA/PO-based samples, were monitored over time by spectroscopic analysis. Furthermore, to complete the preliminary analysis of these encapsulating films, mechanical and rheological characterizations were also performed.

## 2. Materials and Methods

Two different commercial polymers, poly(ethylene-co-vinyl acetate) (EVA) and linear low-density polyethylene (LLDPE), were used to formulate blends with compositions of EVA/LLDPE = 100/0 and 75/25 wt./wt.%, respectively. Poly(ethylene-co-vinyl acetate) (EVA) is a Greenflex**^®^** copolymer (EVA28, Versalis spa, San Donato Milanese, Italy) with the following physical characteristics: vinyl acetate 28%, density = 0.550 g/cm^3^, MFR (190 °C/2.16 kg) = 25 g/10 min, Tm = 75 °C. The linear low-density polyethylene (PO) is a commercial Ziegler–Natta Clearflex**^®^** polyolefin (LLDPE, FG106, Versalis spa., San Donato Milanese, Italy) with the following physical characteristics: density = 0.918 g/cm^3^, MFR (190 °C/2.16 kg) = 1 g/10 min, Tm = 125 °C.

A crosslinking agent (here referred to as CA), stabilizing systems consisting of a UV adsorber, a metal deactivator, a phenolic antioxidant (here referred to as cumulative sigla STAB) and a polar wax, as a promoter of adhesion, were added in different percentages, see Table 1:

The EVA and EVA/PO samples with and without additives were processed in a Brabender mixer (Brabender® GmbH & Co. KG, Duisburg, Germany) at 150 °C for 5 min at 50 rpm. The additives were added following a specific protocol. In particular, the crosslinking agent (CA) was added to the matrices after 4 min of processing and mixed for 1 min; all stabilizers (UVabs, MD and AO) were added to the matrices after 3 min of processing and mixed for 2 min, while the adhesion promoter TEG was added to the matrix pellets before the mixing.

For all characterizations, the EVA and EVA/PO-based samples were obtained as films in a Carver press at 150 °C for 5 min (1 min of preheating and 4 min of pressing). The thermal treatment under high pressure was performed to simulate the thermal treatment experienced by the encapsulant film of the PV modules, i.e., lamination and assembly conditions.

The used characterizations were as follows:

- Accelerated photoageing was performed as follows: Photo-oxidation was carried out using a Q-UV/basic weatherometer (from Q-LAB, Westlake, OH, USA) equipped with UVB lamps (313 nm, intensity 0.9 W/m^2^). The weathering conditions consisted of a continuous light irradiation at T = 70 °C. The irradiation ranged from 0.85–0.9 W/m^2^
- Tensile tests were carried out using a universal testing machine (Instron model 3365, High Wycombe, UK), according to the ASTM D882 method on rectangular films. The tests were performed using a tensile speed of 1 mm/min for 1 min to evaluate the Young′s modulus, and then the velocity was increased to 10 mm/min until a sample breakage.
- Rheological tests were performed using a stress-controlled rheometer (ARES G-2) in a parallel plate geometry (plate diameter of 25 mm). The complex viscosity (η*), storage (G′) and loss (G″) moduli were measured under frequency scans from ω = 10^−1^ to 100 rad/s at T = 150 °C. The strain amplitude was γ = 5%, which preliminary strain sweep experiments proved to be low enough to be in the linear viscoelastic regime.
- A Fourier-transform infrared spectrometer (Spectrum One, Perkin Elmer, Waltham, MA, USA) was used to record the IR spectra using 16 scans at a resolution of 1 cm^−1^. The progress of the photo-oxidation degradation of the samples was followed by FTIR analyses, monitoring the variations of some peak areas in the hydroxyl range (3200–3600 cm^−1^) and the carbonyl range (1800–1600 cm^−1^) in time, using the Spectrum One software.
- A UV–visible Spectrometer, (Specord^®^250 Plus, Analytik Jena, Jena, Germany) was used to record UV–Vis spectra by performing 8 scans between 200 and 1100 nm at a resolution of 1 nm. The values of the linear attenuation coefficient (*k*) were calculated considering the measured absorption values (A) at 750 nm and the sample thickness (D) using the following formula: *k* = A/(2.3 D).

## 3. Results and Discussion

### 3.1. Mechanical Characterization

**Figure 1:**
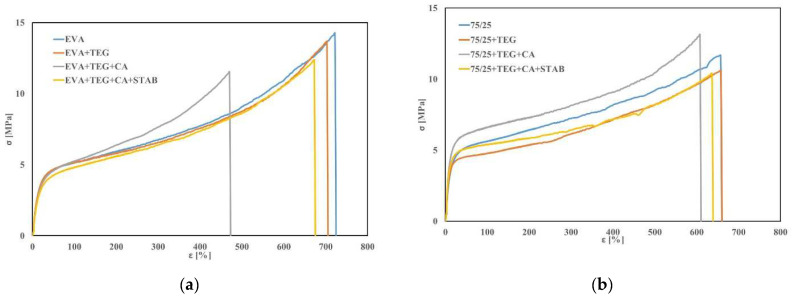
Stress-strain curves of the (**a**) EVA and (**b**) EVA/PO = 75/25 wt.% samples with and without an adhesion promoter (TEG), a crosslinking agent (CA) and stabilizers (STAB).

**Figure 2:**
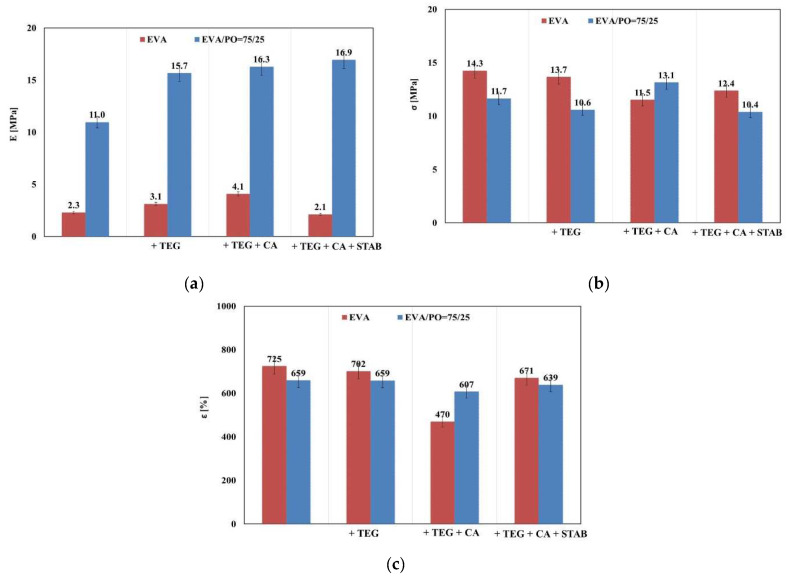
Main mechanical properties. (**a**) Elastic modulus, E; (**b**) tensile strength, σ; (**c**) elongation at break, ε of the EVA and EVA/PO = 75/25 wt.% samples with and without an adhesion promoter (TEG), a crosslinking agent (CA) and stabilizers (STAB).

Good elasticity, reflected through Young′s modulus, and high elongation at break are the desirable mechanical properties of polymeric encapsulants. Here, the mechanical behavior of the analyzed samples was assessed by a monoaxial tensile test. In Figure 1a,b, the stress-strain curves of the EVA and EVA/PO samples with and without additives are shown. In addition, in Figure 2a–c, the trends of the elastic modulus (E), the tensile strength (σ) and the elongation at break (ε) for the systems with different compositions are also shown.

It is noteworthy that the values of the elastic modulus are significantly higher for the sample containing 25 wt.% of PO compared to the pristine EVA, as previously documented in the literature [23]. By adding an adhesion promoter and a crosslinking agent, the modulus values were further increased for all of the investigated samples. This behavior could be attributed to the good anchoring of Tegomer in both polymer matrices; thus, the additional entanglements lead to a modulus increase. Moreover, the crosslinking agent forms chemical bonds to join the polymer chains together, leading to the formulation of stiffer systems.

The stabilizing agents, with their lower molecular weights, have a lubricating effect on the EVA-based system, decreasing the elastic modules and increasing the maximum elongation in comparison to the same composites without the stabilizers. On the contrary, by adding the stabilizers to the EVA/PO-based system (i.e., EVA/PO + TEG + CA + STAB), the sample shows higher values for the elastic modulus, while the elongation and tensile strength at break remain almost unchanged with respect to the system without additives.

It can be underlined that the EVA/PO sample containing all of the additives (i.e., EVA/PO + TEG + CA + STAB) shows an increase of about 58% in the elastic modulus value with respect to the pure system (EVA/PO), while the tensile strength and the elongation at break remain unchanged. It could be said that the stabilizers, in particular, the antioxidants, are efficient against the degradation phenomena occurring upon processing the melt, preserving the mechanical properties [23,24]. Summarizing, the EVA/PO + TEG + CA + STAB is the sample that shows the best mechanical properties, and it is the most suitable material for the polymeric encapsulant with respect to the other investigated samples.

### 3.2. Rheological Characterization

**Figure 3:**
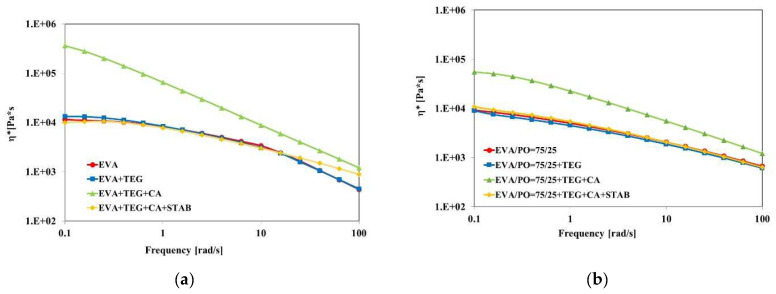
Viscosity curves of the (**a**) EVA and (**b**) EVA/PO = 75/25 wt.% samples with and without an adhesion promoter (TEG), a crosslinking agent (CA) and stabilizers (STAB).

**Figure 4:**
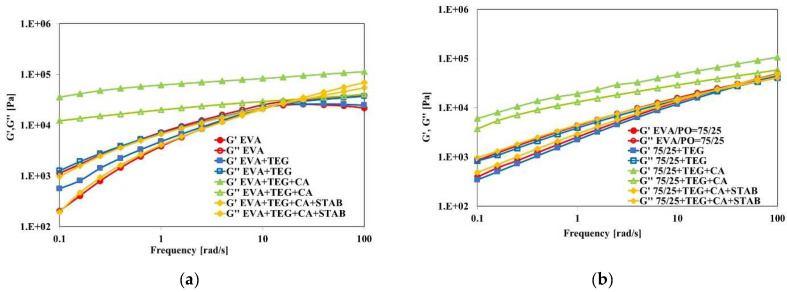
Storage (G′) and loss (G″) moduli of the (**a**) EVA and (**b**) EVA/PO = 75/25 wt.% samples with and without an adhesion promoter (TEG), a crosslinking agent (CA) and stabilizers (STAB).

The results of the performed frequency sweep tests are reported in Figure 3a,b and Figure 4a,b. In particular, Figure 3a,b show the trends of the complex viscosity as a function of the angular frequency. In the EVA + TEG system (Figure 3a), the values of the complex viscosity are almost the same as those of the pristine EVA over the investigated shear rates, indicating that the viscoelastic properties are still dominated by the polymer matrix and no negative plasticizing effect due to the presence of TEG is noticed. At a lower frequency, however, the complex viscosity values are higher in the presence of the adhesion promoter, and this behavior can be explained by considering the good adhesion at the interface between the polymer chains and polar wax molecules. The sample containing the polar wax and the crosslinking agent, i.e., EVA + TEG + CA, exhibits a greater complex viscosity value and pronounced shear thinning phenomena, indicating a non-Newtonian behavior and a pseudoplastic nature because of the crosslinked structure. Nevertheless, when the stabilizing agents were added to the EVA-based system, the complex viscosity values remained close to the values of the EVA matrix by up to 100 rad/s. At a higher angular frequency, the complex viscosity values remained higher than those of the pure matrix, exhibiting diminished frequency dependence. This result can be attributed to the plasticizing effect of the added lower molecular weight molecules of the stabilizers, according to the mechanical analysis.

The same roughly rheological considerations can be inferred for the EVA/PO systems (Figure 3b). The melt rheological behavior of EVA/PO also changes after the incorporation of CA and TEG, highlighting the slightly changed rheological behavior. However, at a lower frequency region (ω < 0.2 rad/s), the sample showed a Newtonian behavior, while the melt rheological behavior of the EVA/PO+TEG+CA+STAB is similar to that of the pure EVA/PO and EVA/PO + TEG.

Therefore, for both EVA and EVA/PO systems, adding CA leads to a significant increase in the viscosity values, especially in the low frequency range, suggesting the formation of crosslinked structures. It is worth noting that the viscosity values for both EVA and EVA/PO upon the addition of CA and TEG are similar to those of neat samples and samples containing only TEG, suggesting good processability for these materials.

In addition, the values of the storage and loss moduli, G′ and G′, for all of the investigated samples are reported in Figure 4a,b. The values of the storage modulus of the EVA + TEG sample exceeded those of the EVA matrix at a frequency lower than 10 rad/s (Figure 4a). Therefore, the G′ and G″ modulus trends for both EVA + TEG + CA (Figure 4a) and EVA/PO + TEG + CA (Figure 4b) showed parallel trends in all of the examined frequency regions, and no crossover points were observed. Moreover, the G′ values are higher as compared to those of the pure EVA and EVA/PO matrices, and they are not very sensitive to the increase in the angular frequency, confirming that the rheological behavior changed from liquid-like to solid-like behavior due to the formation of a three-dimensional crosslinked structure. Finally, it can be seen that the flow curves are not significantly influenced by the presence of the stabilizers.

### 3.3. Spectroscopy Characterization

#### 3.3.1. FTIR Analysis

**Figure 5:**
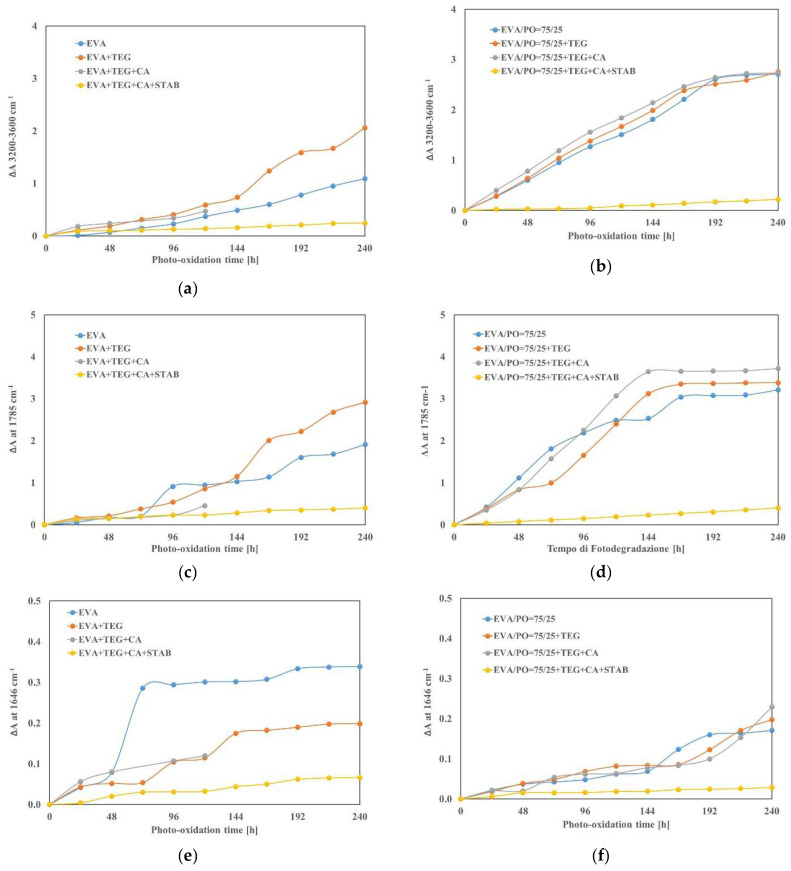
Variation of the peak areas at (**a**,**b**) 3200–3600 cm^−1^, (**c**,**d**) 1785 cm^−1^ (**e**,**f**) and 1646 cm^−1^ for the EVA and EVA/PO = 75/25 wt.% samples with and without an adhesion promoter (TEG), a crosslinking agent (CA) and stabilizers (STAB).

FTIR spectroscopy is a well-known and widely used technique to study the chemical changes occurring in organic materials. Therefore, the degradation process was analyzed by monitoring the spectroscopic changes that occur during the photo-oxidation process. The FTIR spectra, in the range of 4000–450 cm^−1^, were performed on the pure investigated matrices, i.e., EVA and EVA/PO = 75/25 wt.%, and on the samples containing an adhesion promoter (TEG), a crosslinking agent (CA) and stabilizers (STAB) at different exposure times; they are reported as Supplementary Materials in Figures S1–S8. The FTIR analysis of all investigated samples was carried out for more than 800 h, but, here, we reported the analyses of the results obtained in the first 240 h of the accelerated ageing, since this time is significant for the beginning of the photo-oxidative degradation process, see Figure 5a–f.

According to the literature, the characteristic absorption bands of EVA are CH_n_ vibrations in the ranges of 3000–2800 cm^−1^, 1500–1400 cm^−1^ and 1000–700 cm^−1^, and the bands related to the movements of the vinyl acetate components are 1734, 1365 and 1234 cm^−1^ [23,24,25,26]. The thicknesses of all of the considered films are about 450 µm, the same as commercial encapsulant films, also according to the literature [24], and for this reason, some peaks are almost saturated.

The resulting qualitative differences over time in the spectra of the investigated systems were detected in two principal domains, which are the carbonyl domain (1600–1800 cm^−1^) and the hydroxyl domain (3200–3600 cm^−1^). These changes are correlated with the current knowledge of the degradation mechanisms of the materials, including carbonyl formation and related oxidative reactions and hydroxyl production. It was observed that the variations of the absorbance peak in the range of 3200–3600 cm^−1^ broadened after the accelerated weather testing results, see Figure 5a,b. Moreover, the increases in the intensities of the absorption band at 1785 cm^−1^ and 1646 cm^−1^ were also monitored, see Figure 5c–f.

Indeed, according to the literature, the band at 1785 cm^−1^ is due to lactone formation, while the band at 1646 cm^−1^ is attributed to the vinyl alkene vibrations, which are also formed because of the EVA degradation [23,24]. As shown in Figure 5a,b, in the hydroxyl domain, the EVA and EVA/PO samples, such as the related systems containing Tegomer and Tegomer plus crosslinking agent, are more sensitive to the photo-oxidation process, and the EVA + TEG + CA sample was irreversibly deteriorated after only 120 h of ageing. Instead, no important changes in the absorbance variation were observed over time in the following samples containing stabilizers: both EVA + TEG + CA + STAB and EVA/PO + TEG + CA + STAB had to be protected against photodegradation.

The absorbance peaks related to the lactone in the EVA + TEG and the EVA + TEG + CA sharply increased after 96 h (Figure 5c), and this last sample, as said before, failed after 120 h. The EVA matrix began to significantly degrade after 72 h, while for the EVA/PO-based system, except for the stabilizers containing the sample, the sharp increase proceeded after only 24 h (Figure 5d). The stabilizing agents’ presence in the EVA and EVA/PO-based systems leads to a linear trend with a lower growth over time with respect to the other investigated samples.

The results of the FTIR characterization, related to the variation in absorbance of the peak at 1646 cm^−1^, also confirm that the stabilizers extend the chemical stability of the EVA and EVA/PO systems and that better performance is finally observed for the aged EVA/PO + TEG + CA + STAB sample. Therefore, to understand the obtained results, it needs to be considered that the stabilizers (STAB) have overall beneficial effects for both EVA and EVA/PO because they act efficiently for the stabilization of both EVA and PO. The crosslinking agent (CA), being a suitable additive for EVA crosslinking, causes large carbonyl accumulation in polyolefin in the EVA/PO blend, while the commercial Tegomer (TEG) is a suitable adhesion promoter for polymer blends containing polyolefins.

#### 3.3.2. UV-Vis Analysis

The UV-Vis analysis of all investigated samples is very important because, in UV range, the presence of the additives that are able to absorb UV irradiation can be identified, while in the visible range, the transparency ability of encapsulant films can be detected.

**Figure 6:**
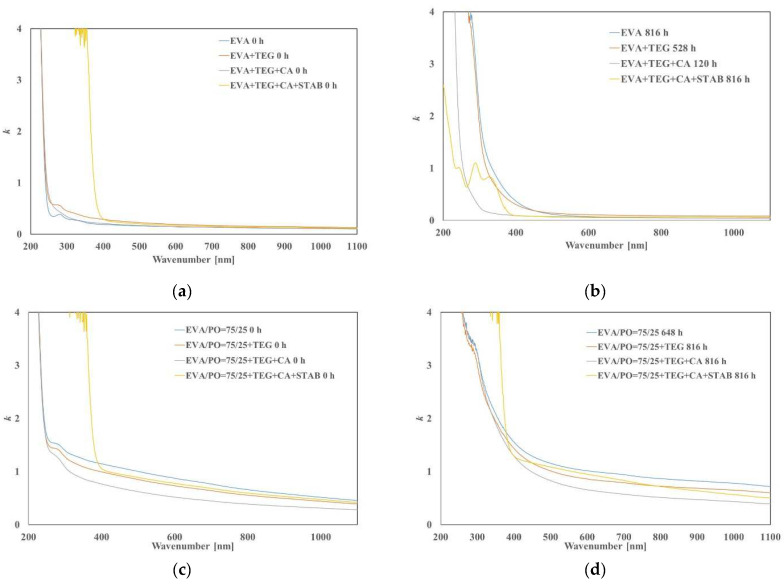
Linear attenuation coefficient (*k*) of EVA (**a**) before exposure and (**b**) at maximum UVB exposure time and of EVA/PO = 75/25 wt.% (**c**) before exposure and (**d**) at maximum UVB exposure time with and without an adhesion promoter (TEG), a crosslinking agent (CA) and stabilizers (STAB).

In Figure 6a–d, the values of the linear attenuation coefficient (*k*) for all investigated samples as a function of the wavelength (200–1100 nm range) at 0 h and after UVB exposure (at the maximum exposure time) are reported. These analyses are carried out to test the optical properties and to evaluate the transparency in the visible range. Indeed, the linear attenuation coefficient (*k*) measures the extent to which the incident light is attenuated when passing through the materials; therefore, the more transparent materials have a *k* value approaching zero.

In the UV region below 400 nm, the *k* values are higher if UV stabilizers are presented because of their absorption ability in the ultraviolet region. The addition of a UV absorber is required for the polymers used for the encapsulations because the films must be protected against active solar radiation (from 295 to 400 nm); otherwise, the adsorbed radiation will induce bond cleavage processes in these materials. The obtained spectra show that increasing the irradiation time in the EVA-based system results in changes in the *k* values, especially before the visible region, i.e., up to 400 nm (Figure 6a). Such behavior may be related to the absorption of the decomposition and/or degradative products. It is also noted that the EVA + TEG + CA + STAB sample shows a lower *k* between 200 and 400 nm after accelerated ageing, with respect to all other samples containing stabilizer systems; this could be attributed to a small amount of UV stabilizers that are lost by diffusion, evaporation or leaching. Anyway, according to the FTIR results, the EVA + TEG + CA + STAB sample maintains an overall chemical structure integrity, but it is worth mentioning that the gradual loss of the stabilizer protection is dangerous because it could affect the rate of degradation and may also cause inhomogeneous degradation. Altogether, the EVA-based systems display lower *k* values than the values of the EVA/PO-based systems, both before and after UVB exposure. The higher *k* values reported for the EVA/PO system in all investigated wavelengths are likely due to the presence of linear low-density polyethylene that adds a little more haze to the sample. Moreover, a higher *k* in the UV region between 430 and 470 nm is indicated by a yellowish color. The aged EVA/PO-based samples exhibit the same trend as the EVA-based samples, showing higher *k* values between 200 and 400 nm, and at major wavelengths, the differences with the unaged samples are less meaningful.

**Figure 7:**
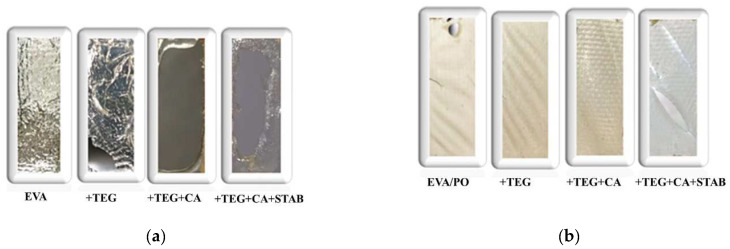
Pictures of the (**a**) EVA and (**b**) EVA/PO = 75/25 wt.% samples with and without an adhesion promoter (TEG), a crosslinking agent (CA) and stabilizers (STAB) at maximum photo-degradation time.

The pictures of all the examined systems at the maximum photo-degradation time are reported in Figure 7. With regard to the EVA/PO-based samples, the images reveal that the samples without stabilizers have noticeably yellowed upon UVB exposure. Instead, the EVA/PO sample containing the appropriate additives, i.e., EVA/PO + TEG + CA + STAB, does not show yellowing, even if it is visibly degraded. The reported pictures highlight and confirm the beneficial effect of the stabilizing system in maintaining the optical properties over time. It is worth noting that the pictures of the EVA samples were obtained using a black-colored background, and that all of these samples were extremely brittle at maximum exposure time.

According to the spectroscopic analyses, we can deduce that the stabilizers enhance the photostability of the EVA and EVA/PO-based systems, and as reported in the literature, they are more efficient in stabilizing the PO than the EVA [23]; a better performance is overall attributed to the EVA/PO + TEG + CA + STAB sample. Finally, both the EVA + TEG + CA + STAB and EVA/PO + TEG + CA + STAB samples can be considered good encapsulants for the production of double-sided photovoltaic modules.

## 4. Conclusions

In photovoltaic plants, each single component must be optimized and designed to be economically advantageous and to work for a long time, and as known, the durability and stability during the operating life must be guaranteed. In the photovoltaic module production process, an employed approach to ensure and increase the operational stability of a solar cell is the use of polymeric encapsulants that prevent environmental degradation. Anyway, for a long outdoor service, all polymeric materials must be protected against photo-oxidation, even in the case when the polymers are not directly exposed to ultraviolet light. As known, the polymers could contain chromophore impurities, such as peroxides and ketones, which absorb light, and the breakdown of the polymeric chains can lead to a premature loss of the material’s properties and performance. Understanding how to improve the durability and efficiency of the modules is becoming a necessity. The internal stresses must be avoided, and the choice of materials, such as additives, and the morphology and compatibility of the materials drive the reliability and durability of PV modules. Therefore, this work supports the importance of optimizing the chemistry of EVA-based materials for obtaining good mechanical performance, extending module lifetimes, as well as preventing discoloration and maintaining transparency and optical properties according to the literature and based on previous results that suggested the benefits of the formulation of an EVA/PO blend for PV encapsulant films and the use of a silane-free adhesion promoter. The reported results established that the silane-free polar wax Tegomer could be successfully used as an adhesion promoter because it could preserve the delamination of the photovoltaic modules caused by silane degradation during the operating life that, indeed, accelerates the degradative processes. Moreover, a performing polymeric encapsulant may be formulated by adding UV stabilizers and antioxidants to minimize the net extent of the photo-oxidation degradation, which is caused by photothermal stresses. Indeed, the mechanical and rheological behaviors and the optical properties of both EVA and EVA/PO films containing different additives (TEG + CA + STAB) are very similar, suggesting good processability and performance for both encapsulants. These preliminary analyses carried out on the films outline the beneficial effects of the used adhesion promoter for the encapsulating polymeric EVA/PO films and indicate that the samples EVA + TEG + CA + STAB and EVA/PO + TEG + CA + STAB can be considered as good encapsulants for double-sided photovoltaic modules.

## Figures and Tables

**Table 1:** Commercial name, producer and chemical formula of used materials.

Additive, Used Sigla and Commercial Name	Producer	Chemical Formula	Notes
Crosslinking agent (CA); Luperox^®^ 101 (LUP)	Sigma-Aldrich	2,5-bis(tert-butylperoxy)-2,5-dimethylhexane	CA was added at 1.5 wt.% during processing
ultraviolet light absorber (UV abs); Chimassorb^®^ 81	Ciba Specialty Chemicals	methanone, [2-hydroxy-4-(octyloxy)phenyl]phenyl	UVabs was added at 1 wt.% during processing
Metal deactivator and antioxidant (MD); Irganox^®^ MD 1024	Ciba Specialty Chemicals	2′,3-bis[[3-[3,5-di-tert.-butyl-4-hydroxyphenyl]propionyl]] propionohydrazid	MD was added at 0.5 wt.% during processing
Primary phenolic antioxidant (AO); Irganox^®^ 1076	Ciba Specialty Chemicals	octadecyl 3-(3,5-di-tert-butyl-4-hydroxyphenyl) propionate	AO was added at 0.5 wt.% during processing
polar wax (TEG), Tegomer^®^ E 525	Evonik Goldschmidt Italia, s.r.l.	TEG was used with the aim of promoting adhesion between the layers of material. It is a fine-grained white powder with a melting point close to 100 °C and a melt viscosity of 200 MPa s to 140 °C. It has been added at 2.5 wt.% before processing	

## Data Availability

Not applicable.

## Supplementary Materials

The following supporting information can be downloaded at: https://www.mdpi.com/article/10.3390/ma15196751/s1, Figure S1. FTIR spectra of EVA at different exposure times; Figure S2. FTIR spectra of EVA+TEG at different exposure times; Figure S3. FTIR spectra of EVA+TEG+CA at different exposure times; Figure S4. FTIR spectra of EVA+TEG+CA+STAB at different exposure times; Figure S5. FTIR spectra of EVA/PO=75/25 wt.% at different exposure times; Figure S6. FTIR spectra of EVA/PO=75/25 wt.% +TEG at different exposure times; Figure S7. FTIR spectra of EVA/PO=75/25 wt.% +TEG+CA at different exposure times; Figure S8. FTIR spectra of EVA/PO=75/25 wt.% +TEG+CA+STAB at different exposure times.
