# Supplementary material for: Polar Wax as Adhesion Promoter in Polymeric Blend Films for Durable Photovoltaic Encapsulants
Source: Materials (Basel). 2022 Sep 29;15(19):6751. doi: 10.3390/ma15196751 (PMC9571350; doi:10.3390/ma15196751)
Supplement: Supplementary file 1 [file materials-15-06751-s001.zip › materials-1892428-supplementary.pdf]

Supplementary Information

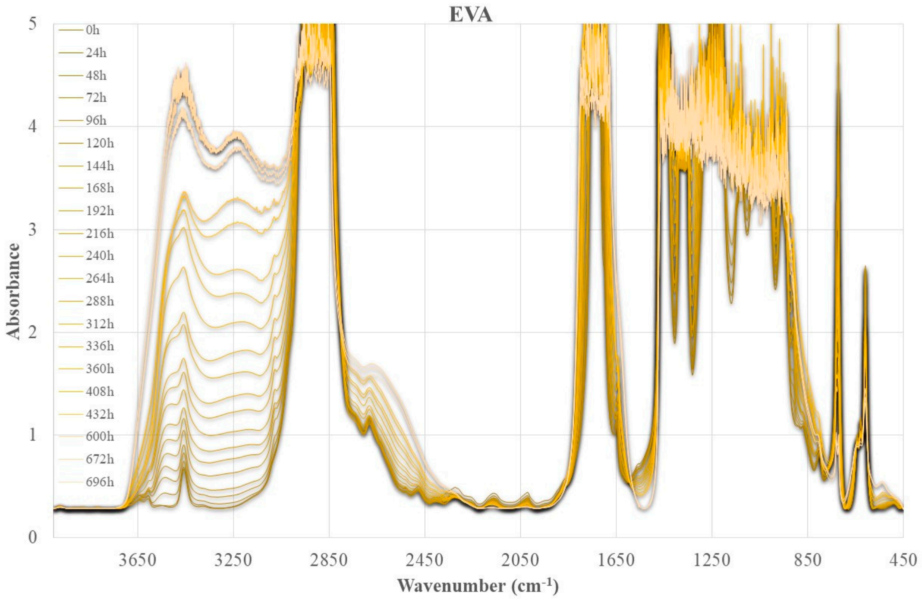


Figure S1. FTIR spectra of EVA at different exposure times

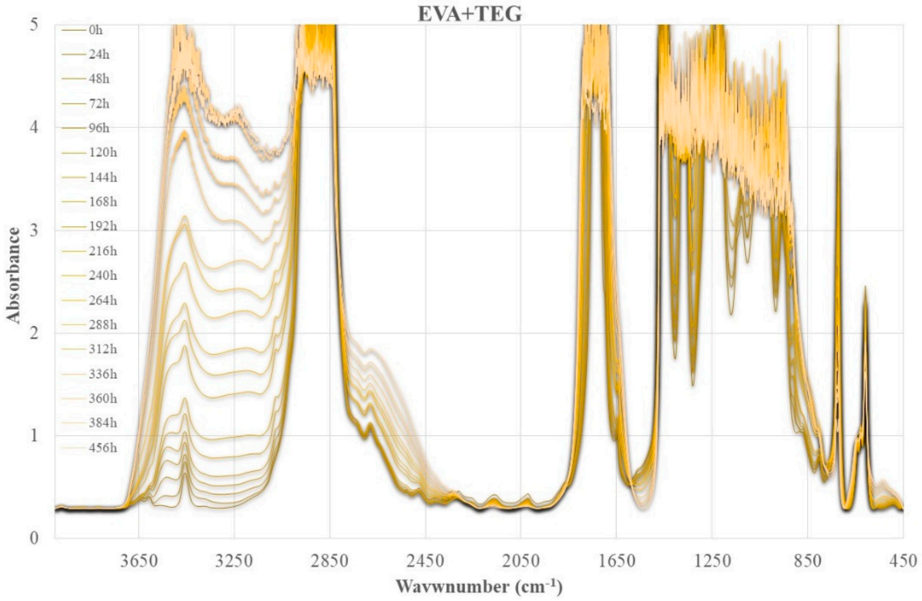


Figure S2. FTIR spectra of EVA+TEG at different exposure times

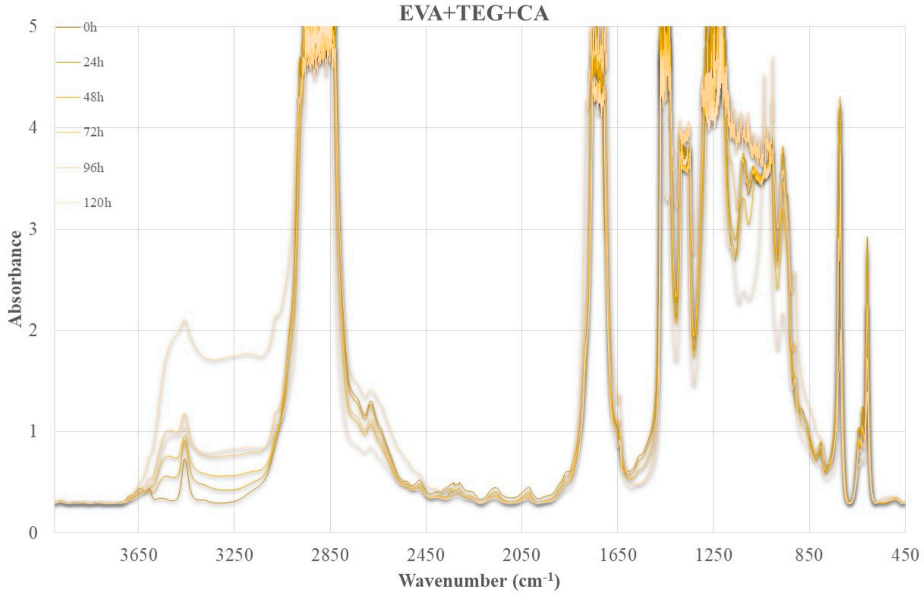


Figure S3. FTIR spectra of EVA+TEG+CA at different exposure times

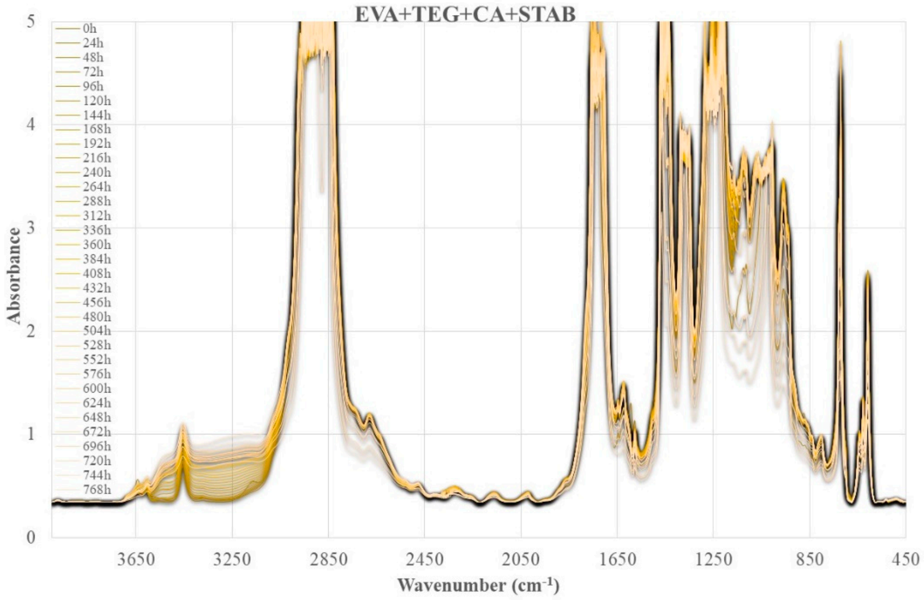


Figure S4. FTIR spectra of EVA+TEG+CA+STAB at different exposure times

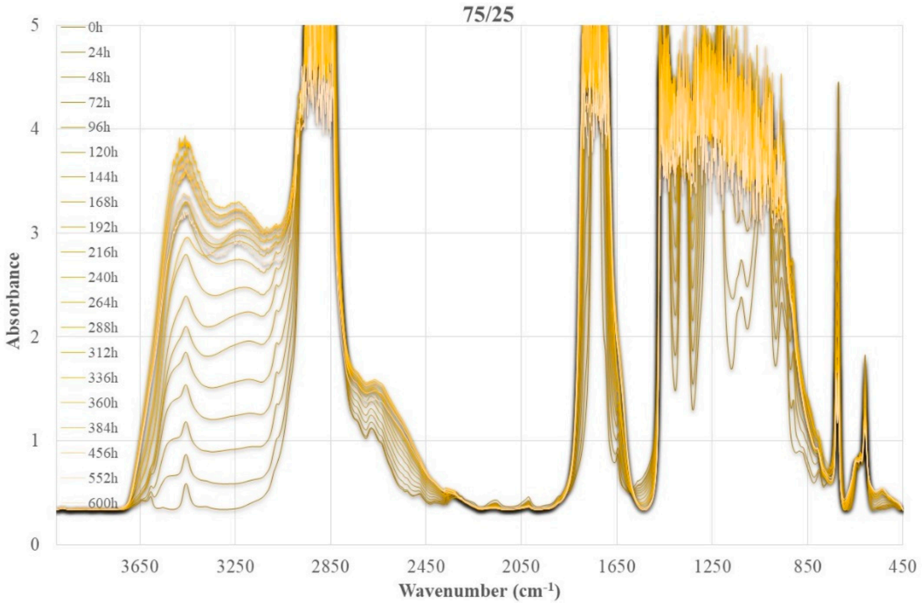


Figure S5. FTIR spectra of EVA/PO=75/25 wt.% at different exposure times

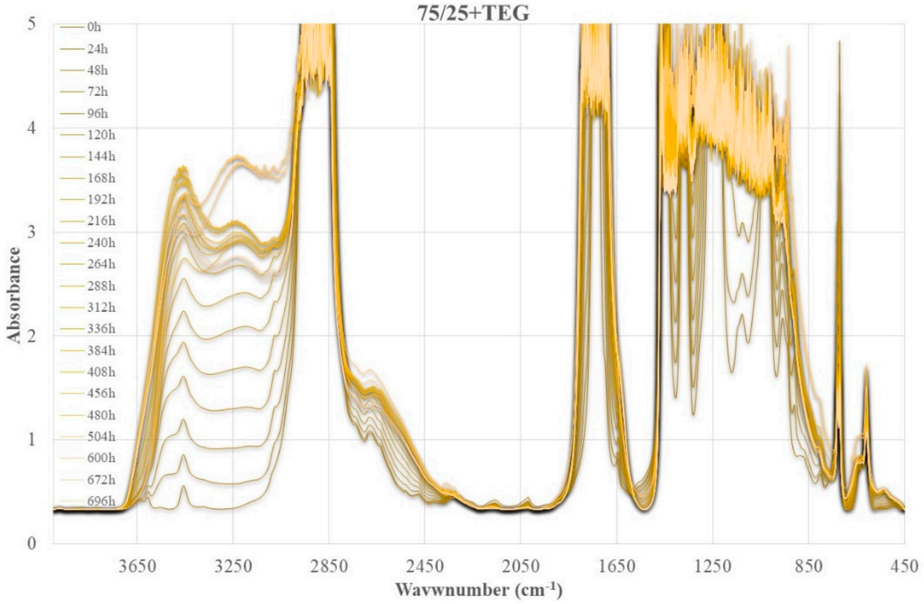


Figure S6. FTIR spectra of EVA/PO=75/25 wt.% +TEG at different exposure times

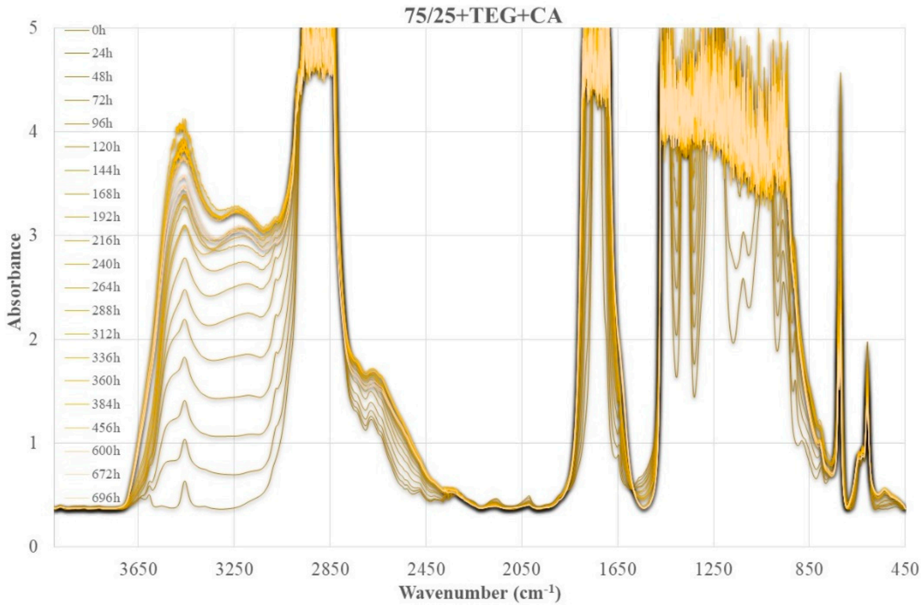


Figure S7. FTIR spectra of EVA/PO=75/25 wt.% +TEG+CA at different exposure times

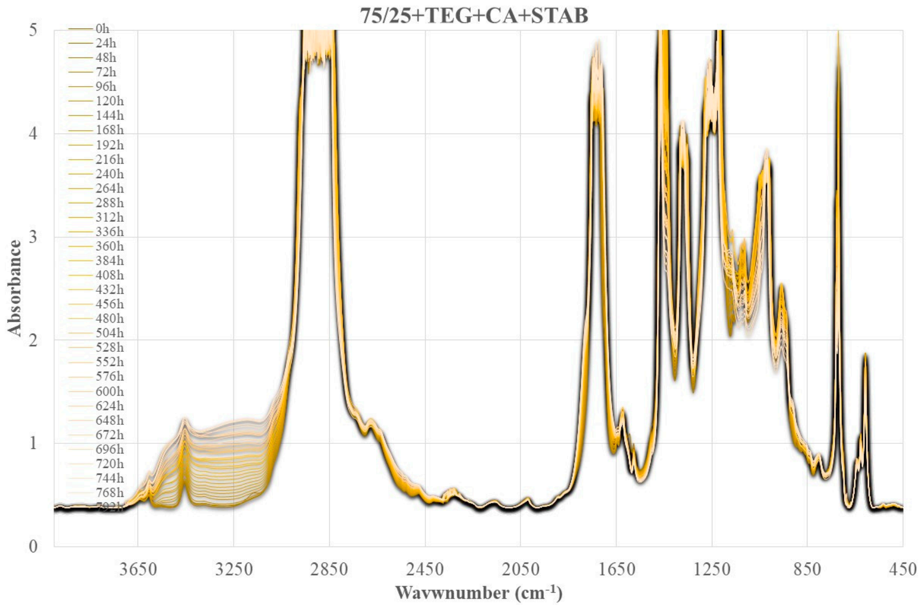


Figure S8. FTIR spectra of EVA/PO=75/25 wt.% +TEG+CA+STAB at different exposure times
